# Targeting NEDD8 suppresses surgical stress-facilitated metastasis of colon cancer via restraining regulatory T cells

**DOI:** 10.1038/s41419-023-06396-6

**Published:** 2024-01-05

**Authors:** Yi Jiang, Shenjia Gao, Hao Sun, Xinyi Wu, Jiahui Gu, Han Wu, Yun Liao, Ronen Ben-Ami, Changhong Miao, Rong Shen, Jinlong Liu, Wankun Chen

**Affiliations:** 1 Department of Anesthesiology, Zhongshan Hospital, Fudan University, Shanghai, 200032 China; 2 Shanghai Key laboratory of Perioperative Stress and Protection, Shanghai, 200032 China; 3 School of Basic Medical Science, Shanghai Medical College of Fudan University, Shanghai, 200032 China; 4 Infectious Diseases Unit, Tel Aviv Sourasky Medical Center, Faculty of Medicine, Tel Aviv University, Tel Aviv, 6997801 Israel; 5 Department of Pathology, Nanfang Hospital, Southern Medical University, Guangzhou, 510515 Guangdong China; 6 Institute of Translational Medicine, Shanghai University, Shanghai, 200444 China; 7 Department of Anesthesiology, Shanghai Geriatric Medical Center, Shanghai, 201104 China; 8 Department of Anesthesiology, QingPu Branch of Zhongshan Hospital, Fudan University, Shanghai, 201799 China

**Keywords:** Cell death and immune response, Experimental models of disease

## Abstract

Regulatory T cells (Tregs) are a key determinant for the immunosuppressive and premetastatic niche for cancer progression after surgery resection. However, the precise mechanisms regulating Tregs function during surgical stress-facilitated cancer metastasis remain unknown. This study aims to unravel the mechanisms and explore potential strategies for preventing surgical stress-induced metastasis by targeting NEDD8. Using a surgical stress mouse model, we found that surgical stress results in the increased expression of NEDD8 in Tregs. NEDD8 depletion abrogates postoperative lung metastasis of colon cancer cells by inhibiting Treg immunosuppression and thereby partially recovering CD8^+^T cell and NK cell-mediated anti-tumor immunity. Furthermore, Treg mitophagy and mitochondrial respiration exacerbated in surgically stressed mice were attenuated by NEDD8 depletion. Our observations suggest that cancer progression may result from surgery-induced enhancement of NEDD8 expression and the subsequent immunosuppressive function of Tregs. More importantly, depleting or inhibiting NEDD8 can be an efficient strategy to reduce cancer metastasis after surgery resection by regulating the function of Tregs.

## Introduction

Surgery resection remains the first-line treatment for solid malignancies. However, patients are still at high risk of developing cancer recurrence and metastatic diseases even after the complete resection [1]. It has been acknowledged that surgical stress itself facilitates cancer metastasis, during which the suppression of anti-tumor immunity contributes to a major part [2]. Regulatory T cells (Tregs) play a critical role in repressing anti-tumor effector cells such as cytotoxic T cells and natural killer (NK) cells, thereby exacerbating the postoperative immunosuppression and the formation of premetastatic niche. The amplification and activation of Tregs under surgical stress have been observed [3]. Our previous study also demonstrates that surgical stress induces cancer recurrence and metastasis by promoting Treg recruitment and function [4]. However, limited literature has investigated the regulatory mechanisms by which surgical stress regulates Tregs and the resulting metastasis of cancer cells.

Neddylation is the process of posttranslational protein modification catalyzed by E1 activating enzyme, E2 conjugating enzyme, and E3 ligase, which dominated by neuronal precursor cell-expressed developmentally down-regulated protein 8 (NEDD8), a ubiquitin-like protein conjugates and activates the E3 ligase [5]. Canonical neddylation refers to the binding and activation of Cullin-RING E3 ligases (CRLs) by NEDD8, leading to substrate degradation [6]. However, several substrates of NEDD8 other than the Cullin molecules have been identified in recent years, and the process is known as non-canonical neddylation. Proteins encountered non-canonical neddylation can have diverse fates, such as increased stability, increased enzymatic activity, changes in subcellular location, or degradation [7]. Since neddylated proteins undergo structural modification and altered biochemical and functional properties [8], NEDD8 can participate in multiple vital biological events via neddylation modification.

Dysregulated expression of NEDD8 and aberrant neddylation is found associated with diseases such as neurodegenerative disorders [9], ischemic brain injury [10], and tumors [11]. In particular, the neddylation pathway has been found overactivated in multiple human cancers, such as liver cancer [12], lung cancer [13] and colorectal cancer [14], leading to accelerated degradation of tumor suppressors and hence the progression of tumors. Moreover, neddylation blockade has been found of potential therapeutic value for cancer metastasis [15]. Neddylation has also emerged as a critical mechanism in regulating immune cell function, such as the proliferation and migration of macrophages [16]. Recent studies disclosed that neddylation E2 Ube2m-E3 Rbx1 axis is essential for the maintenance of Treg functionality [17, 18]. Overexpressed UBA3, the sole catalytic subunit of NEDD8-activating enzyme E1, was associated with the recruitment and infiltration of Tregs in lung adenocarcinoma [19]. However, little has been known about how exactly NEDD8 regulates the function of Tregs. It has been found that Neddylation can be induced in response to various cellular stresses, including proteasomal impairment, heat shock, and oxidative stress [20]. However, whether it responds to surgical stress is unknown.

In the current study, we investigated the role of NEDD8 in surgical stress-facilitated cancer metastasis and the involvement of Tregs, and revealed the underlying mechanisms. NEDD8 was found upregulated in Tregs upon surgical stress. Depletion of NEDD8 significantly ameliorated the postoperative lung metastasis of colon cancer cells in mice. During this time, mitophagy and mitochondrial respiration activated in Tregs by surgical stress were restrained by NEDD8 blockade. These data demonstrate that the increased expression of NEDD8 in Tregs contributes to the postoperative lung metastasis of colon cancer cells via the immunosuppressive function of Tregs, blocking NEDD8 can partially recover the anti-tumor immunity to suppress surgical stress-facilitated metastasis.

## Methods

### Clinical samples

This study was approved by the Ethics Committee of Fudan University Shanghai Cancer Center (FUSCC), China (Protocol #1901208-13), and complied with the ethical standards set out in the Declaration of Helsinki. The exclusion criteria included previous or other concomitant cancers, received preoperative radiotherapy, chronic inflammatory diseases, autoimmune diseases, and a loss of contact during follow-up. From January 2020 to December 2020, 20 patients undergoing colorectal cancer surgery were recruited for the study. All participants provided written informed consent. Peripheral blood was collected in sampling tubes containing EDTA. Clinical data regarding patient characteristics and surgical procedures were recorded. The patients were followed up after surgery every 3 months.

### Animal model

C57BL/6 wild-type (WT, NEDD8^+/+^) female mice (8 weeks old) and NEDD8 knock-out (NEDD8^−/−^) female mice were purchased from Shanghai Laboratory Animal Research Center (Shanghai, China). To analyze the endogenous role of NEDD8 expression in Tregs, we generated a cell-type-specific conditional knock out mouse. This mouse was bred by mating NEDD8^fl/fl^ mice with Foxp3^Cre^ mice to specifically deplete NEDD8 on Tregs (NEDD8^fl/fl^-Foxp3^Cre^). The NEDD8^fl/fl^ mice and Foxp3^Cre^ mice were both purchased from Shanghai Laboratory Animal Research Center (Shanghai, China).

Animals were housed in specific pathogen-free conditions under a 12-hour light-dark cycle at the Department of Laboratory Animal Science of Fudan University. All animal experiments were performed in accordance with the guidelines of the ethics committee on animal experimentation of Zhongshan Hospital, Fudan University (protocol license number: 2020-119).

Surgical stress model was induced in mice as previously described with modification [21]. Briefly, mouse colon cancer cell line MC38 was purchased from the American Type Culture Collection (ATCC) and cultured in DMEM (Gibco) supplemented with 10% FBS (Gibco). Mice were intravenously injected with MC38 cells (1 × 10^6^ cells) in 100 mL of PBS. Surgery stress were then induced by abdominal laparotomy (3 cm midline incision) and left nephrectomy to establish pulmonary metastases. Surgery commenced 10 minutes following tumor inoculation. In some experiments, MC38 cells were labeled with CFSE [22] before injection. At 3 weeks post-surgery, mice were sacrificed, whole blood, lung tissues, and spleens were harvested for further experiments. For lymphocytes purification, lymph nodes were harvested. In some experiments, to further determine the effect of surgery on the in vivo metastasis of MC38 cells, CFSE-labeled MC38 cells in murine lungs 6 h, 12 h, and 24 h post-surgery were detected by flow cytometry and immunofluorescence.

### Histological examination and immunohistochemistry

For tumor metastases assessment, both lungs were gently dissected and photographed for quantification of lung tumor burden. For histological examination, formalin-fixed paraffin-embedded lung tissue was cut into 4- μM sections, placed on glass slides, dewaxed with xylene, and rehydrated with 30–100% ethanol. The sections were microwave-boiled in extract buffer (10 mM citric acid buffer, pH 6.0) for 5 min and stained with H&E, and a histopathological examination was performed. The stained sections were analyzed under a light microscope (Carl Zeiss). For immunofluorescence experiment, pulmonary specimens were fixed with 4% paraformaldehyde and embedded in optimal cutting temperature compound before sectioned. Frozen sections were incubated with DAPI (Invitrogen). Immunofluorescence was assessed by immunofluorescence microscopy (Olympus, Tokyo, Japan), and data were collected with ImageJ (Bethesda).

### Enzyme-linked immunosorbent assay (ELISA)

Murine whole blood was centrifuged at 3000 RPM for 15 min, and serum was collected and stored at −80 °C. Serum concentrations of IFN-γ, TNF-α, IL-6, IL-10, IL-17, CCL-17, IL-4, IL-10, IL-13, TGF-β were determined using ELISA kits according to manufacturer’s instructions (R&D, USA). A total of 50 µL serum was used for one cytokine analysis. The optical density (OD) at a wavelength of 450 nm was measured using the Infinite F50® microplate reader manufactured by Tecan (CH).

### Flow cytometry

Peripheral blood mononuclear cells (PBMCs) and splenocytes were isolated from murine blood and spleens by Ficoll density gradient cell separation using Histopaque 1083 (Sigma-Aldrich) and red blood cell lysis. Tumor-infiltrating lymphocytes (TILs) in lung tissue were isolated by Ficoll-Pague (GE Healthcare) density gradient centrifugation. Single-cell suspensions of the PBMCs, TILs, and splenocytes of mice were prepared. Cells were suspended in FACS buffer (eBioscience) and incubated with mouse Fc blocker to block nonspecific binding sites. For surface molecules staining, cells were incubated in the dark with fluorochrome-conjugated antibodies specific for cell surface antigens for 30 minutes at 4 °C and fixed with 1% PFA. For intracellular staining, cells were stimulated with brefeldin A for 6 hours, and cell surface antigens were labeled with fluorochrome-conjugated antibodies. Cells were then permeabilized and fixed in fixation/permeabilization solution (eBioscience), washed with permeabilization buffer (eBioscience), and then incubated for 30 minutes at 4 °C in the dark with intracellular antibodies against cytokine, chemokines, or transcriptional factors. Stained cells were analyzed using FACSVerse (BD), and data files were analyzed using FlowJo software (Tree Star).

### CD8^+^ T cell cytotoxicity assay

LDH cytotoxicity assay was performed to assess the cytotoxicity of CD8^+^T cell against colon cancer cells MC38. Normally LDH is abundant in the cytoplasm and cannot pass through the cell membrane normally, but can be released outside the cell when the cell is damaged or dies, and the released LDH can be detected by enzymatic reaction in the medium supernatant. Purified CD8^+^T cells were obtained from the spleens and lymph node of MC38 tumor-bearing mice using CD8a^+^T cell Isolation Kit (Miltenyi Biotec) as effector cells. MC38 cells were used as target cells. A total of 200 μL of effector cells and target cells were assigned at different E:T ratios to each well of 96-well plate and incubated for 4 h at 37 °C. Lactate dehydrogenase (LDH) release was subsequently assessed by incubation of the supernatants with the provided substrate for 30 min, and the absorbance was read at 490 nm. Activity was calculated using the formular: % cytotoxicity = (experimental effector_spontaneous_−target spontaneous/target_maximum_−target spontaneous) × 100, as described previously [23].

### NK cell cytotoxicity assay

Ex vivo NK cell cytotoxicity was measured by the Calcein-AM assay as previously described [24] to assess the tumor-killing function of NK cells against colon cancer cells MC38. Briefly, NK cells (NK1.1^+^) were purified from spleens of MC38 tumor-bearing mice using NK Cell Isolation Kit (Miltenyi Biotec). NK cells were then re-suspended and stimulated with 50 U/ml IL-2 for expansion and activation before incubated with Calcein-AM (Invitrogen)-labeled MC38 cells at different ratios incubated for 4 h at 37 °C. Target tumor cells in the absence of NK cells were used for detection of spontaneous release. Target cells added with lysis buffer were used for detection of max lysis. After 4 h, cells were centrifuged and supernatants of cell mixture were harvested for detection. The reading values of fluorescence intensity (FI) in the supernatants were measured at excitation 495 nm and emission 515 nm using a PerkinElmer Victor X3 Microplate Reader (Perkin Elmer). Activity was calculated using the formula: % cytotoxicity = [FI (sample wells)−FI (spontaneous release)] × 100/[FI (max release)−FI(spontaneous release)].

### Treg suppressive activity assay

Suppressive activity assay of Tregs was performed as previously described [25]. Briefly, Tregs were purified from spleens of MC38 tumor-bearing mice using CD4^+^CD25^+^ Regulatory T Cell Isolation Kit (Miltenyi Biotec). CD8^+^T cells were isolated from spleens of MC38 tumor-bearing mice and labeled with CFSE as target cells. Tregs were co-cultured with CD8^+^T cells in 96-well plate at different ratio in addition with 2μg/ml anti-CD3 plus anti-CD28. CD8^+^T cells in the absence of Treg cells were used for detection of the count of loaded CD8^+^ T cells. Cells were harvested and the proliferation of target cells were assessed by flow cytometry. Treg suppressive activity was calculated using the formular: % suppression = Count (CD8^+^T cells) *100/Count (control CD8^+^T cells).

### Proliferation and cell viability assay

Ki67 assay was conducted to determine the proliferation of Tregs. Briefly, Tregs were stained with Ki67-Alexa Fluor 488 and detected by flow cytometry. CCK8 assay was used to determine the cell viability of Tregs. Briefly, Tregs were planted in 96-well plate and incubated with anti-CD3 plus anti-CD28 for 24 h, 10 μl CCK8 were added and cultivated at room temperature for 4 h. Absorbance was measured at 450 nm. In some experiments, cells were treated with mitophagy inducer carbonyl cyanide 3-chlorophenylhydrazone (CCCP), mitochondrial division inhibitor 1 (Mdivi-1), or vehicle (DMSO).

### RNA-seq

RNA-seq was processed by Shanghai Biochip Co., Ltd., (Shanghai, China) according to the instructions of NEB Next Ultra RNA Library Prep Kit for Illumina (New England Bio Labs). Briefly, total RNAs were isolated from Tregs purified from spleens of wild-type mice subjected to sham (animals underwent anesthesia without aparotomy and nephrectomy) or laparotomy surgery using Trizol reagent. Poly(A) RNA was subsequently purified and used to generate cDNA libraries. All samples were sequenced on Illumina HiSeq X Ten platform. Sequence reads were mapped to the human genome version hg38 by using Illumina sequence analysis pipeline. The average gtranene expression values of three independent studies were used for analysis. Volcano plot, GO analysis, and KEGG pathway analysis were performed.

### Mitophagy detection

The morphology changes of mitochondria in Tregs were analyzed by transmission electron microscope (TEM). Mito-Tracker Green (Cot #M7514, Invitrogen) was used in the immunofluorescence assay for visualizing mitochondria. Mitochondrial DNA (mtDNA) copy number was determined by real-time PCR. Relative mitochondrial membrane potential in Tregs was measured by JC-1 assay kit (Cot # C2006, Beyotime). ATPase activity in Tregs was measured by ATPase Assay Kit (Cot # ab234055, Abcam). ROS content in Tregs was measured by DCFDA/H2DCFDA–Cellular ROS Assay Kit (Cot # ab113851, Abcam). All experiments were conducted according to the manufacturer’s instructions. In some experiments, cells were treated with mitophagy inducer CCCP, mitochondrial division inhibitor 1 (Mdivi-1), or vehicle (DMSO).

### Oxygen consumption rate (OCR) measurement

To assess the mitochondrial respiration in Tregs, OCR was measured using the Seahorse Cell Mito Stress Test Kit (Cot # 103010-100, Agilent) according to the manufacturer’s instructions as previously described [26]. In some experiments, cells were treated with mitophagy inducer CCCP, mitochondrial division inhibitor 1 (Mdivi-1) or vehicle (DMSO).

### Neddylation assay kit

Neddylation Assay Kit (ab139468, Abcam) was used to determine the level of neddylation in Tregs according to the manufacturer’s instructions. This kit provides the means of generating a thioester-linked NEDD8-conjugated E2 enzyme, utilizing the first two steps in the NEDD8 cascade, for use in the NEDDylation of E3 ligases and target substrate proteins UbcH12. A NEDD8-specific antibody is provided for detection of NEDDylated proteins via SDS-PAGE and western blotting. Western blot of biotinylated-NEDD8 (Cot # BML-UW0560) thioester assay. Bt-NEDD8 conjugated to UbcH12 (Cot # BML-UW9145).

### Co-immunoprecipitation (Co-IP) and GST pull-down assay

Co-immunoprecipitation (Co-IP) and GST pull-down assay were conducted as previously described [27, 28] to determine the protein interaction between NEDD8 and Parkin. Co-IP assay of NEDD8 and Parkin from 293 T cells and murine Tregs, either over-expressing NEDD8 (OE-NEDD8) or treated with neddylation inhibitor MLN4924 (3 μM) was conducted. Western blot analysis of whole-cell lysates and immunoprecipitates with indicated antibodies. For GST pull-down, The Nedd8 CDS sequence was inserted into the pGEX-4T-1 vector (Amersham Biosciences). Parkin was inserted into the pET-28a vector (Novagen). To detect direct binding, bacteria-expressed GST-tagged proteins were immobilized on glutathione Sepharose 4B beads (Amersham Biosciences) and then incubated with His-tagged proteins for 8 h under rotation. Beads were washed with GST-binding buffer (100 mM NaCl, 50 mM NaF, 2 mM EDTA, 1% NP-40, and protease inhibitor mixture), and proteins were eluted, followed by immunoblotting.

### Western blotting

Protein was isolated from tissues or cells, separated by 12% SDS-PAGE, and transferred to PVDF membranes. Membranes were then probed with primary antibodies against target genes at 4 °C overnight, followed by incubating with HRP-conjugated anti-mouse secondary antibodies at room temperature for 1 hour.

### Quantitative real-time PCR

Total RNA was extracted from murine tissues or cells by TRIzol reagent (Invitrogen, USA), and cDNA was extracted by an ABI High-Capacity cDNA Reverse Transcription Kit (Thermo Fisher Scientific, Waltham, MA, USA) according to the manufacturers’ instructions. Quantitative real-time PCR was performed using the ABI StepOnePlus Real-Time PCR system (Applied Biosystem, Foster City, CA, USA). GAPDH was used as an endogenous control. Data were analyzed using 2^- ΔΔCT^.

### Antibodies and primers

Antibodies and primers used in this study were listed in Supplementary Tables 1 and 2.

### Statistical analysis

The experimental data were statistically analyzed by SPSS 17.0 software. All experiments were repeated at least three times independently. Quantitative data are expressed as the means ± standard error of the mean (SEM). The mean of two independent samples was compared by Student’s *t* test, one-way and Two-way analysis of variance (ANOVA) was used for multi-group comparisons followed by Bonferroni post-hoc analysis. Differences were considered significant at *P < 0.05; **P < 0.01; ***P < 0.001; ****P <  0.0001.

## Results

### Surgical stress increases the expression of NEDD8 in Tregs

**Fig. 1:**
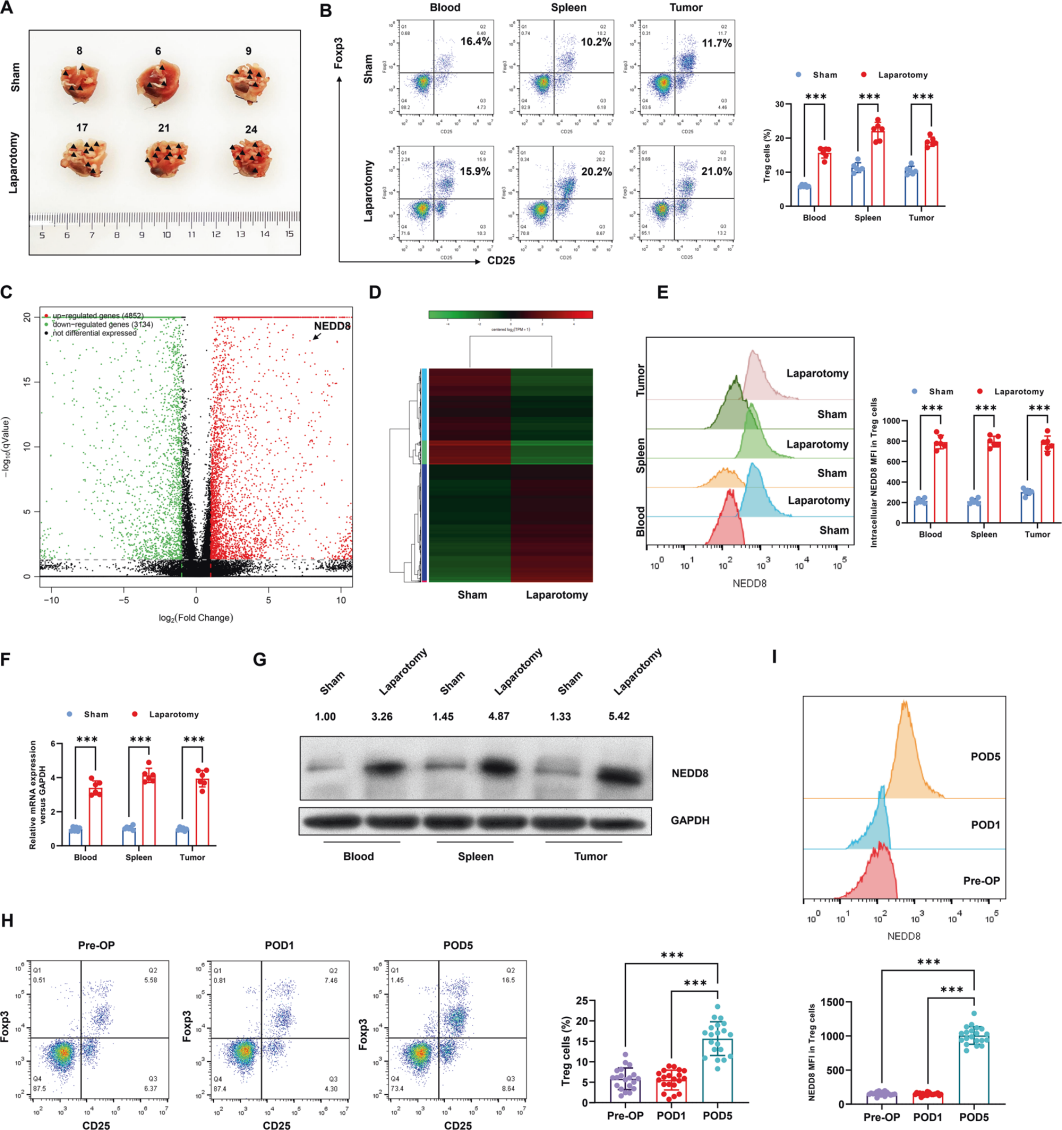
Surgical stress increases the expression of NEDD8 in Tregs. Wild-type mice were randomly subjected to sham or laparotomy surgery after the inoculation of MC38 cells (*n* = 6 per group). Blood, spleens, and metastatic pulmonary tumors were harvested on D3 post-surgery. Tregs were isolated from murine blood, spleens, and tumors. **A** Representative images of the metastatic foci on murine lung surfaces. Black arrows indicate the metastatic foci on the photo. We observed 8, 6, 9 metastatic foci on the lung surface of three sham mice while the numbers were 17, 21, 24 in the laparotomy group. **B** The percentages of Treg (CD3^+^CD4^+^Foxp3^+^) cells in blood, spleens, and tumors were detected by flow cytometry. **C**, **D** RNA-seq of Tregs from spleens were conducted. **C** Volcano plot with the log2 fold changes in gene expression after laparotomy surgery on the *x* axis and the statistical significance (−log10 *p* value) on the *y* axis. **D** Differential expressed genes after laparotomy surgery were shown in heatmap. **E** Flow cytometry was performed to determine the positive percentage of intracellular NEDD8 in Tregs in blood, spleens, and tumors. The mRNA and protein expression of NEDD8 in Tregs were detected by **F** real-time PCR and **G** western blot, respectively. **H**, **I** Peripheral blood was collected from colorectal cancer patients pre-operatively (Pre-OP), 1 and 5 days post-surgery (POD1, POD5), *n* = 20. The percentages of Tregs in PBMC **H** and the expression of NEDD8 on Tregs **I** were detected by flow cytometry. All experiments were repeated for three times. *P < 0.05, **P < 0.01, ***P < 0.001, ****P < 0.0001.

First, we observed pulmonary metastasis in our surgical stress mice model (Fig. 1A), as well as the increased number of Tregs from blood, spleens, and tumors after laboratory surgery (Fig. 1B). On this basis, this model was utilized to explore the regulatory network of Treg-mediated postoperative metastasis. Tregs were isolated from spleens of mice subjected to laparotomy surgery or sham operation, and RNA-seq was conducted to identify differentially expressed genes in response to surgical stress. Among which, as we can see in the volcano plot (Fig. 1C) and the heatmap (Fig. 1D) of differential expressed genes after laparotomy surgery, NEDD8 was one of the significantly upregulated genes in Tregs of mice underwent laparotomy surgery. It was further verified by the higher levels of mRNA and protein expression of NEDD8 in Treg from laparotomy mice, compared to those from sham-operated mice (Fig. 1E–G). Furthermore, we also detected the enhanced expression of NEDD8 in PBMC from colorectal cancer patients after surgery (Fig. 1H, I). Therefore, it suggests that surgical stress increases the expression of NEDD8 in Tregs.

### Loss of NEDD8 suppresses surgical stress-facilitated lung metastasis of colon cancer cells

**Fig. 2:**
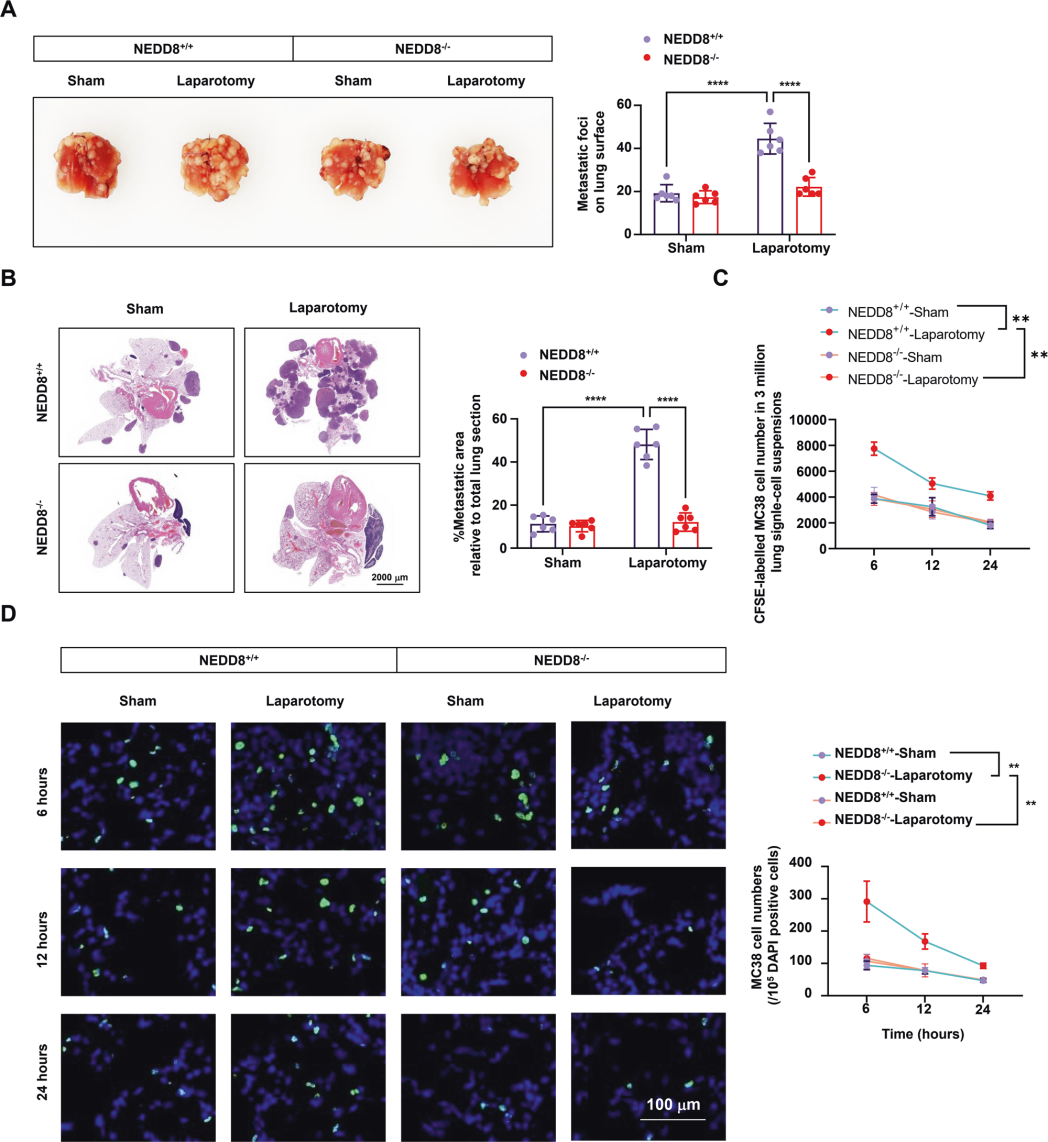
NEDD8^−/−^ mice showed diminished postoperative pulmonary metastasis of colon cancer cells. NEDD8^+/+^ and NEDD8^−/−^ mice were randomly subjected to sham or laparotomy surgery after the inoculation of CFSE-labeled MC38 cells (*n* = 6 per group). **A** Representative images of the metastatic foci on murine lung surfaces on D3 and the statistical data of all mice in each group. **B** Representative images of the H&E staining of the murine lungs on D3 and the calculation of the percentage of metastatic area relative to total lung section (*n* = 6 per group). **C** The number of CFSE-labeled MC38 cells in murine lungs were detected by flow cytometry showing the MFI of CFSE 6 h, 12 h, and 24 h post MC38 injection (*n* = 6 per group). **D** CFSE-labeled MC38 cells in murine lungs 6 h, 12 h, and 24 h post MC38 injection were detected by immunofluorescence (*n* = 6 per group). Green: CFSE, blue: DAPI. Representative images were shown. All experiments were repeated for three times. *P < 0.05, **P < 0.01, ***P < 0.001, ****P < 0.0001.

**Fig. 3:**
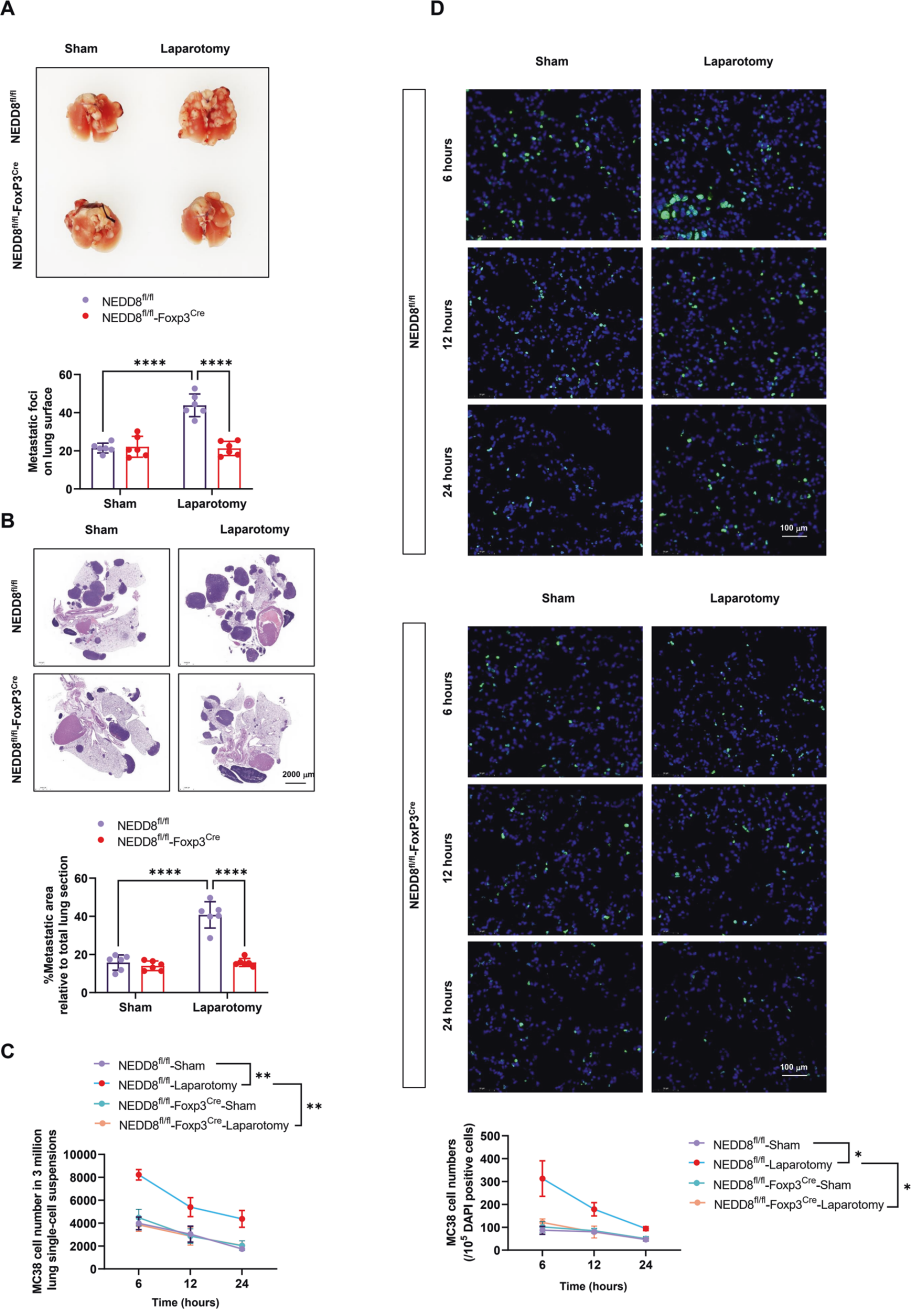
Treg-specific NEDD8 depletion ameliorated postoperative pulmonary metastasis of colon cancer cells in mice. NEDD8^fl/fl^ and Treg-specific NEDD8 depletion (NEDD8 ^fl/fl^-Foxp3^Cre^) mice were randomly subjected to sham or laparotomy surgery after the inoculation of CFSE-labeled MC38 cells (*n* = 6 per group). **A** Representative images of the metastatic foci on lung surface on D3 and the statistical data of all mice in each group. **B** Representative image of the H&E staining of the murine lungs on D3 and the calculation of the percentage of metastatic area relative to total lung section. **C** The number of CFSE-labeled MC38 cells in murine lungs were detected by flow cytometry at 6 h, 12 h, and 24 h post sham or laparotomy surgery. **D** CFSE-labeled MC38 cells in murine lungs 6 h, 12 h, and 24 h post sham or laparotomy surgery were detected by immunofluorescence. Green: CFSE, blue: DAPI. Representative images were shown. All experiments were repeated for three times. *P < 0.05, **P < 0.01, ***P < 0.001, ****P < 0.0001.

To investigate whether NEDD8 functions in surgical stress-induced metastasis, NEDD8 knock out mice was applied for the surgical stress model. For detection of in vivo lung metastasis of colon cancer cells, MC38 cells labeled with CFSE were inoculated during the establishment of model. Postoperative lung metastasis of colon cancer cells was compared between wild-type and NEDD8 knock out mice. Pulmonary metastatic foci profoundly increased upon laparotomy surgery in wild-type mice which have not been observed in NEDD8 knock out mice (Fig. 2A, B). CFSE-labeled MC38 cells were detected to determine the effect of NEDD8 on the in vivo lung metastasis of colon cancer cells. As shown, 6 h, 12 h, and 24 h post inoculation, more newly injected CFSE-labeled MC38 cells were found accumulated in the lungs of wild-type mice with laparotomy surgery than the sham group, but the number remained the same in both groups of the NEDD8 knock out mice (Fig. 2C, D). We then established cell-type-specific deletion of NEDD8 in Tregs. Consistent with the results from NEDD8 knock out mice, Treg specific NEDD8 depletion results in fewer number of metastatic focis on lung surfaces upon laparotomy surgery (Fig. 3A, B), and less infiltration of newly injected CFSE-labeled MC38 cells in the lungs (Fig. 3C, D). Together, these data show that the loss of NEDD8 reduces the postoperative lung metastasis of colon cancer cells in mice and imply the participants of NEDD8 in stress-facilitated cancer metastasis via regulating Tregs.

### Treg expansion induced by surgical stress is impaired by NEDD8 knock out

**Fig. 4:**
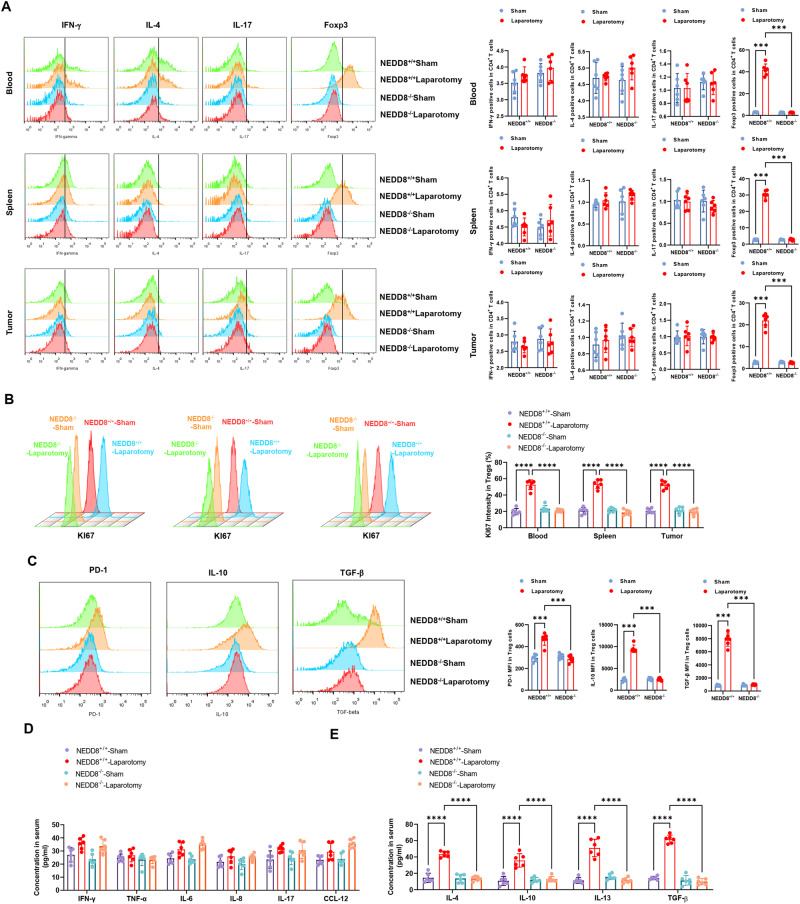
Treg expansion upon surgical stress was reduced by NEDD8 knockout. NEDD8^+/+^ and NEDD8^−/−^ mice were randomly subjected to sham or laparotomy surgery after the inoculation of MC38 cells (*n* = 6 per group). Serum, blood, spleens, and metastatic pulmonary tumors were harvested on D3 post-surgery. PBMC, splenocytes, and tumor-infiltrating lymphocytes were isolated and stained with different molecules to indicate Th cells. **A** The CD3^+^ CD4^+^ T cells were firstly gated, and the percentages of Th1 (CD3^+^CD4^+^IFN-γ^+^), Th2 (CD3^+^CD4^+^IL-4^+^), Th17 (CD3^+^CD4^+^IL-17^+^) and Treg (CD3^+^CD4^+^Foxp3^+^) cells in blood, spleens, and tumors were detected by flow cytometry. **B** Ki67 assay was conducted in Tregs to determine the proliferation. **C** Splenic Tregs were stained with antibodies of PD-1, IL-10, and TGF-b and analyzed by flow cytometry. Serum concentration of pro-inflammatory **D** and anti-inflammatory **E** cytokines were detected by ELISA. All experiments were repeated for three times. *P < 0.05, **P < 0.01, ***P < 0.001, ****P < 0.0001.

Given that NEDD8 in Tregs was increased upon surgical stress, we then examined the effect of NEDD8 knock out on Tregs to investigate how NEDD8 regulates Tregs during surgical stress-induced cancer metastasis. When quantifying lymphocytes in blood, spleens, and tumors, we found that unlike wild-type mice, the percentages of Tregs did not rise after laparotomy surgery in NEDD8 knock out mice (Fig. 4A). The surgical stress-induced proliferation of Tregs was also hampered by NEDD8 knock out (Fig. 4B), accompanied by the defective expression of immunosuppressive transcriptional factors and cytokines (Fig. 4C). It suggests that Treg expansion induced by surgical stress is inhibited by NEDD8 knock out. Tregs play direct roles in shaping cancer immunosuppressive microenvironment by inhibiting anti-tumor cells, including tumor-killing CD8^+^T cells and NK cells. Meanwhile, while the serum concentrations of pro-inflammatory cytokines remained unchanged (Fig. 4D), the levels of of anti-inflammatory cytokines IL-4/10/13 and TGF-β increased after laparotomy surgery in wild-type mice, which however, did not happen in NEDD8 knock out mice (Fig. 4E). These results indicate that the cytokine response was specific for anti-inflammatory cytokine, likely mediated by the action of Tregs. Altogether, it is suggested that NEDD8 is essential for Treg-mediated immunosuppression, which is adverse to cancer metastasis.

### NEDD8 knock out promotes anti-tumor immunity by inhibiting Treg immunosuppression

**Fig. 5:**
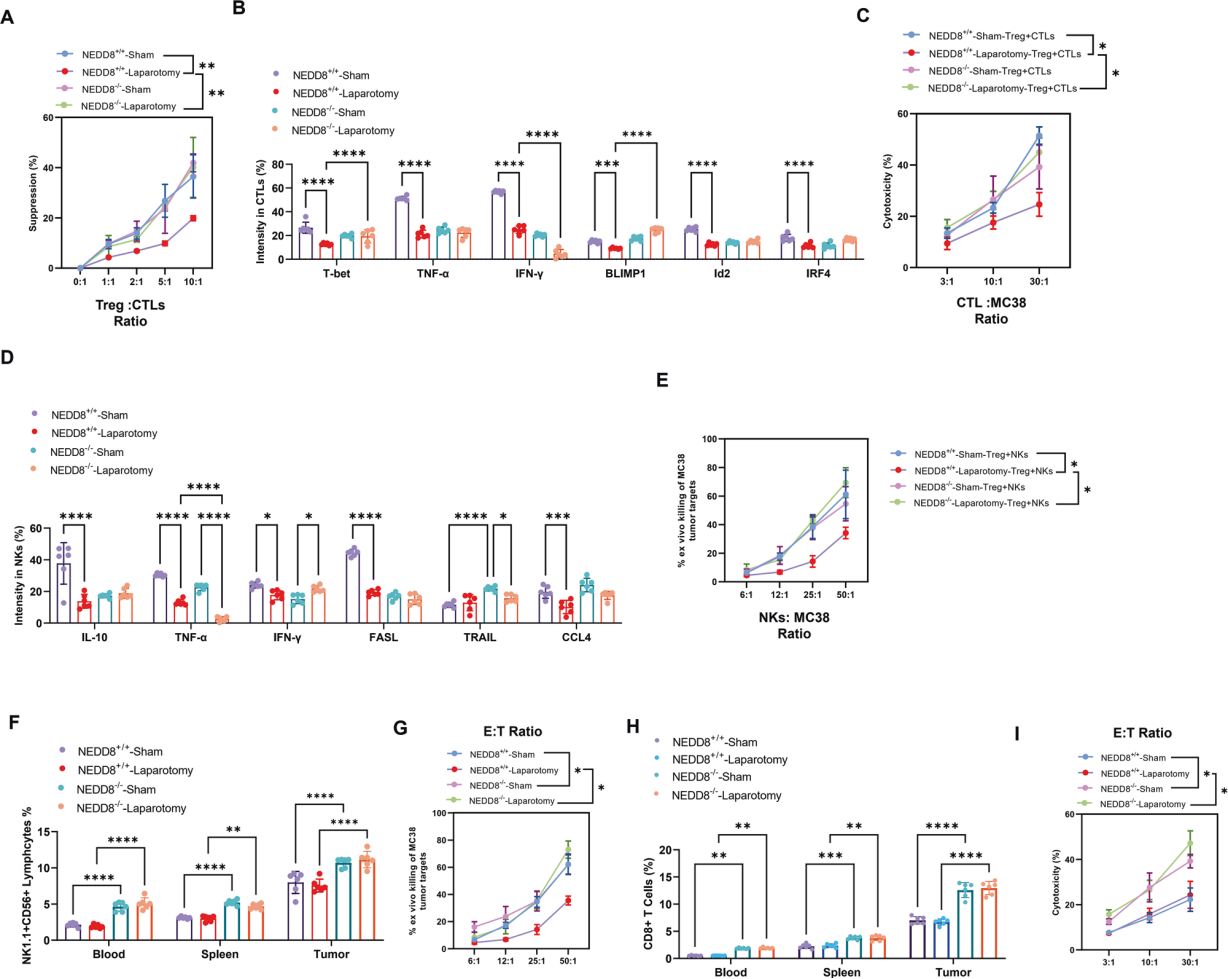
NEDD8 knock out inhibited Treg immunosuppression after surgery. NEDD8^+/+^ and NEDD8^−/−^ mice were randomly subjected to sham or laparotomy surgery after the inoculation of MC38 cells (*n* = 6 per group). Blood, spleens, and metastatic pulmonary tumors were harvested on D3 post-surgery. **A** In vitro suppression assay was performed to determine the ability of splenic Treg in inhibiting the proliferation of cytotoxic CD8^+^T cells (CTLs) upon anti-CD3/CD28 stimulation. CTLs: CD8^+^T isolated from spleens of wild-type MC38 tumor-bearing mice. **B** Splenic Tregs from different groups of mice were co-cultured with CTLs, and flow cytometry was used to detect the expression of cytokines in CTLs after co-culture. **C** After co-cultured with splenic Tregs, CTL assay was conducted to determine the cytotoxicity of CD8^+^T cells on colon cancer cells. **D** NK cells were isolated from wild-type MC38 tumor-bearing mice and stimulated with 50 U/ml IL-2 for expansion and activation. Splenic Tregs from different groups of mice were co-cultured with NK cells. Flow cytometry was used to detect the expression of functional molecules in NK cells after co-culture. **E** After co-cultured with splenic Tregs, ex vivo NK cell cytotoxicity assay was conducted to determine the tumor-killing function of NK cells on colon cancer cells. **F** The percentage of NK cells (NK1.1^+^CD56^+^) cells in blood, spleens, and tumors was determined by flow cytometry. **G** Ex vivo NK cell cytotoxicity assay was conducted to determine the tumor-killing capability of NK cells. E: effector cells, NK cells; T: target cells, MC38. **H** The percentage of CD8^+^T cells in blood, spleens, and tumors was determined by flow cytometry. **I** CD8^+^T cells were isolated from murine spleens and CTL assay was conducted to determine the cytotoxicity of CD8^+^T cells on colon cancer cells MC38. E: effector cells, CD8^+^T cells; T: target cells, MC38. All experiments were repeated for three times. *P < 0.05, **P < 0.01, ***P < 0.001, ****P < 0.0001.

To address the role of NEDD8 in Tregs and the anti-tumor immunity during surgical stress-induced metastasis, Treg immunosuppressive function was determined. In vitro suppression assay was performed to determine the ability of splenic Tregs in inhibiting the proliferation of cytotoxic CD8^+^T cells (CTLs) upon anti-CD3/CD28 stimulation. Unsurprisingly, we observed a higher degree of inhibition of Tregs on the proliferation of CD8^+^T cells after laparotomy surgery, which however, counteracted by NEDD8 knock out (Fig. 5A). To evaluate the direct effect of Tregs on CD8^+^T and NK cell-mediated anti-tumor immunity, CD8^+^T cells and NK cells were co-cultured with Tregs from different groups of mice, respectively. After co-culturing with Tregs of wild-type mice subjected to laparotomy surgery, the expression of functional cytokines and molecules of CD8^+^T cells significantly decreased, compared to the group co-cultured with Treg of sham mice, which however, remained high in NEDD8 mice (Fig. 5B). Same trends were observed in NK cells (Fig. 5D). As measured by cytotoxicity assay, CD8^+^T cells, and NK cells presented better anti-tumor cytotoxicity against colon cancer cells after co-culturing with Tregs of NEDD8 knock out mice, compared to those of wild-type mice (Fig. 5C, E). Furthermore, CD8^+^T cells and NK cells showed distinctly higher levels in NEDD8 knock out mice than in wild-type mice, both in the sham and laparotomy groups (Fig. 5F, H). More importantly, the impaired cytotoxic activity of CD8^+^T cells and NK cells against colon cancer cells after laparotomy surgery was recovered by NEDD8 depletion (Fig. 5G, I). Therefore, these data indicate that NEDD8 knock out impairs the suppressive function of Tregs on CD8^+^T and NK cells and hence partially recovers their anti-tumor activities in the postoperative environment, which restrains cancer metastasis.

### Mitophagy activation in Tregs upon surgical stress is attenuated by NEDD8 knock out

**Fig. 6:**
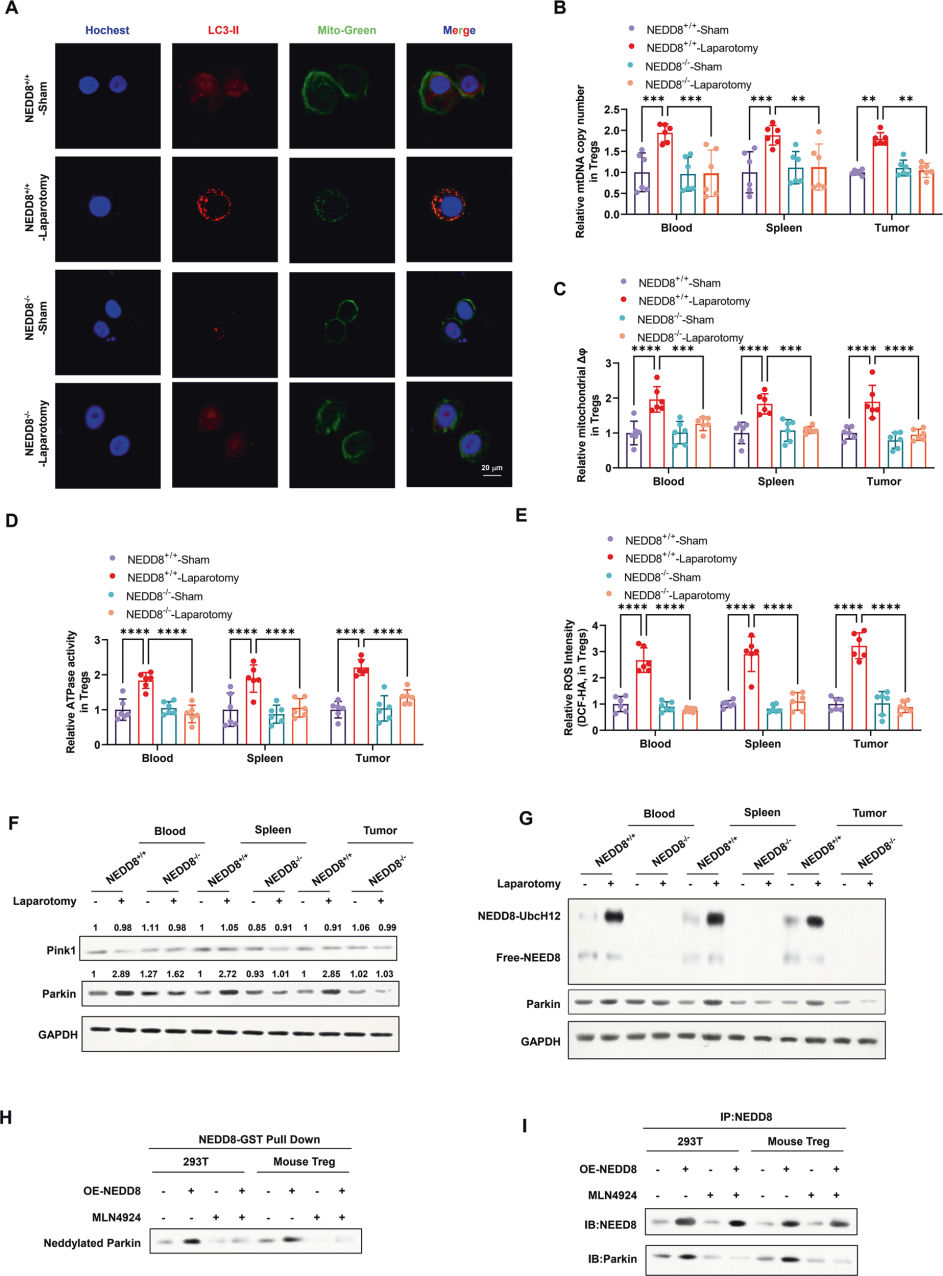
NEDD8-regulated surgical stress-induced Treg mitophagy by Parkin neddylation. NEDD8^+/+^ and NEDD8^−/−^ mice were randomly subjected to sham or laparotomy surgery after the inoculation of MC38 cells (*n* = 6 per group). Blood, spleens, and metastatic pulmonary tumors were harvested on D3 post-surgery and Tregs were isolated. **A** Mitochondria in Tregs isolated from tumors were indicated by Mito-Green in the immunofluorescence assay. Blue: Hotchest, red: LC3-II, Green: Mito-Green. **B** Relative mitochondrial DNA (mtDNA) copy number in Tregs were determined by real-time PCR. **C** Relative mitochondrial membrane potential in Tregs were measured by JC-1 assay kit. **D** ATPase activity in Tregs were measured by ATPase Assay Kit. **E** ROS content in Tregs were measured by DCFDA/H2DCFDA - Cellular ROS Assay Kit. **F** The protein expression of mitophagy-related molecules in Tregs were detected by western blot. **G** Neddylation Assay Kit was used to determine the level of neddylation in Tregs. Protein levels of Free-NEDD8, human Ubc12 (UbcH12) conjugated- NEDD8 (NEDD8-UbcH12), and Parkin was determined by western blot. **H** GST pull-down assay was conducted in 293 T cells and murine Tregs over-expressing NEDD8 (OE-NEDD8) or treated with neddylation inhibitor MLN4924. **I** The protein interaction between NEDD8 and Parkin was verified by Co-IP assay in 293 T cells and murine Tregs, either over-expressing NEDD8 (OE-NEDD8) or treated with neddylation inhibitor MLN4924. All experiments were repeated for three times. All experiments were repeated for three times. **P < 0.01, ***P < 0.001, ****P < 0.0001.

Mitophagic abnormality is closely related to the functional defects of Tregs [29], whether it contributes to postoperative metastasis is unknown. To investigate the change in mitochondrial homeostasis in Tregs upon surgical stress and NEDD8 knock out, morphology of mitochondria and the expression of LC3 was detected. As shown, laparotomy surgery significantly induced higher level of mitophagy in Tregs compared to the sham group (Fig. 6A), along with the enhanced mtDNA content (Fig. 6B), increased level of mitochondria membrane potential (Fig. 6C), ATPase activity (Fig. 6D) and ROS intensity (Fig. 6E). However, mitophagy activation was eliminated by NEDD8 knock out (Fig. 6A). Similarly, the lower mtDNA content indicating the suppressed biogenesis of the mitochondria was also observed in NEDD8 knock out mice (Fig. 6B), as well as the lower levels of indicators of mitochondrial function, the mitochondria membrane potential (Fig. 6C), ATPase activity (Fig. 6D) and ROS intensity (Fig. 6E). These data suggested that NEDD8 depletion attenuated the excessively activated mitophagy in Tregs upon surgical stress.

When evaluating the expression of mitophagy-related proteins, Parkin was found to increase in Tregs in response to surgical stress, but the alteration could not be observed when NEDD8 was depleted (Fig. 6F). Interestingly, Parkin has been identified as a target of NEDD8-mediated neddylation [30]. As the major NEDD8 E2 enzyme, Ubc12 conjugates activated NEDD8 and mediates its subsequent linkage to its target proteins [31]. In wild-type mice, we observed the apparent rise of Ubc12 conjugated-NEDD8 while a decrease of unconjugated/free-NEDD8 in Tregs upon laparotomy surgery, suggesting the emergence of NEDD8-mediated neddylation (Fig. 6G). Together with the moderate-higher protein expression of Parkin (Fig. 6G), it indicates the possibility that Parkin might be the target of neddylation mediated by NEDD8. To further prove the hypothesis, we then examined whether NEDD8 interacted with Parkin in Tregs. The result of GST pull-down assay conducted in 293 T cells showed that NEDD8 could pull-down Parkin, but impaired by neddylation inhibitor MLN4924 (Fig. 6H). In the co-immunoprecipitation experiment, NEDD8 could co-immunoprecipitate with Parkin, further indicating the protein interaction between NEDD8 and Parkin in Tregs (Fig. 6I). Altogether, these data suggest that NEDD8 can enhance mitophagy in Tregs via Parkin neddylation and therefore, NEDD8 depletion attenuated Treg mitophagy with the loss of Parkin neddylation.

### NEDD8-regulated mitophagy confers Treg function and mitochondrial respiration

**Fig. 7:**
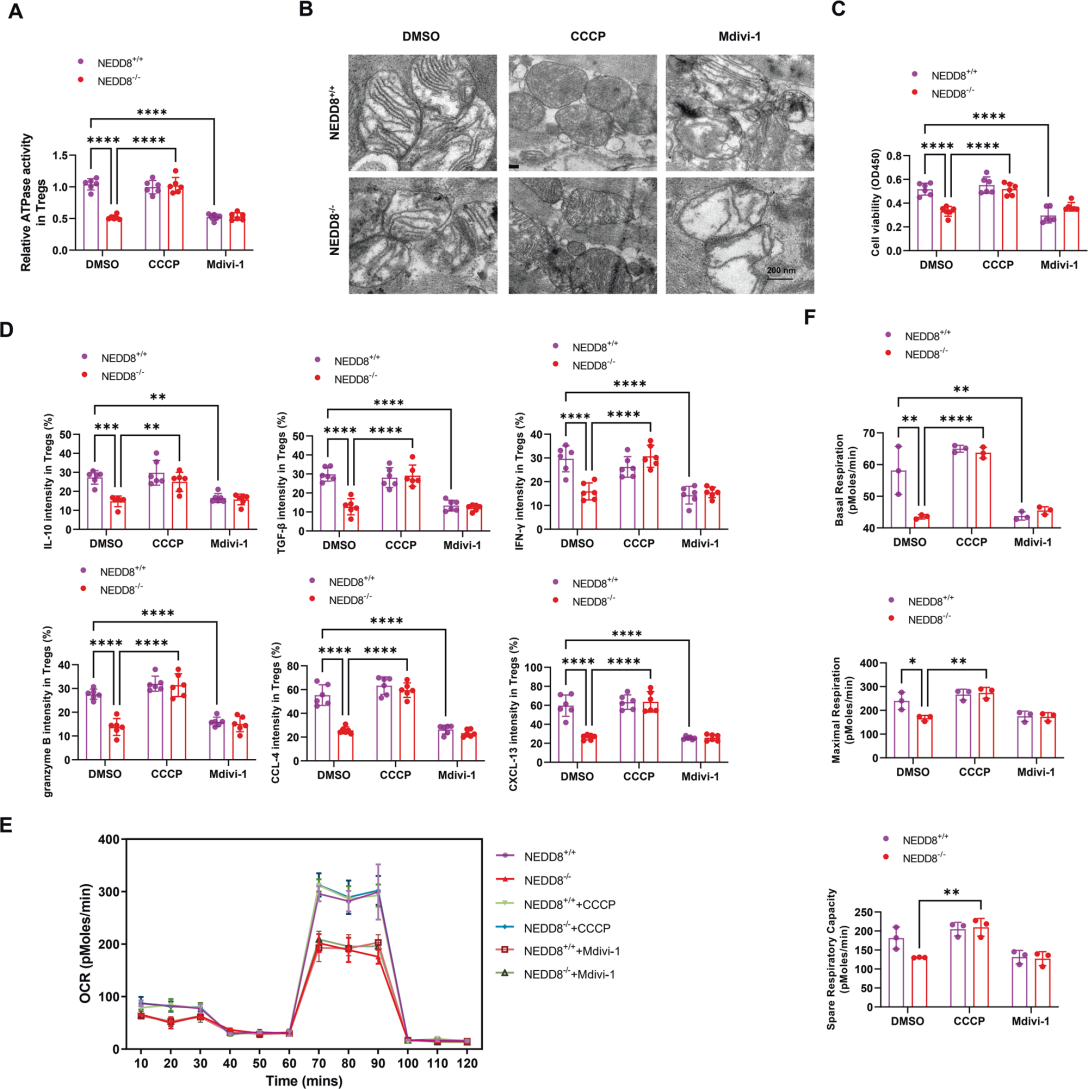
NEDD8-regulated mitophagy conferred Treg function and mitochondrial respiration. NEDD8^+/+^ and NEDD8^−/−^ mice were randomly subjected to laparotomy surgery after the inoculation of MC38 cells (*n* = 6 per group). Tregs were isolated form metastatic pulmonary tumors on D3 post-surgery and were treated with mitophagy inducer CCCP, mitochondrial division inhibitor 1 (Mdivi-1) or vehicle (DMSO). **A** The ATPase activity of Tregs was evaluated. **B** The morphology changes of mitochondria in Tregs were observed by TEM. **C** CCK8 Assay was used to determine the cell viability of Tregs. **D** The intensity of various critical protein expression in Tregs were detected by flow cytometry. **E**, **F** The mitochondrial respiration was analyzed by Cell Seahorse Mito Stress assay. *P < 0.05, **P < 0.01, ***P < 0.001, ****P < 0.0001.

As shown above, surgical stress-induced Treg mitophagy was diminished by NEDD8 depletion. To further support this finding, we modulated mitophagy in vitro in Tregs with or without NEDD8 with mitophagy inducer CCCP or mitochondrial division inhibitor 1 (Mdivi-1). It was observed that mitophagy inducer CCCP rescued the inhibited Treg mitophagy upon NEDD8 knock out, as shown by the recovered ATPase activity of Tregs (Fig. 7A) and the morphology changes of mitochondria (Fig. 7B). While as expectedly, mitophagy of Tregs treated with Mdivi-1 remained low (Fig. 7A, B). Then, we investigated whether mitophagy contributes to the regulatory effects of NEDD8 on Treg function. It was found that NEDD8 knockout hampered the cellular viability of Tregs (Fig. 7C). Moreover, the expression of molecules and cytokines critical for the suppressive function were inhibited (Fig. 7D), suggesting the disrupted immunosuppressive function of Tregs. These were rescued when mitophagy was induced by CCCP, while no significant changes were found in NEDD8 knock-out group when mitophagy was inhibited by Mdivi-1 (Fig. 7C, D). To get a deeper insight into whether Treg functional changes are accompanied by altered mitochondrial respiration, mitochondrial respiratory parameters, including basal and maximal respiration, and spare respiratory capacity were evaluated in Tregs. We found that mitochondrial respiration was restrained by NEDD8 depletion or inhibition (Fig. 7E, F). However, all these effects were compromised when mitophagy or mitochondrial respiration was inhibited. Altogether, these demonstrate that mitophagy regulated by NEDD8 contributes to the alterations in Treg function, as well as mitochondrial respiration.

In conclusion, our data strongly imply that NEDD8 is a major driver of Treg suppressive function induced by surgical stress, predominantly through activating mitophagy. NEDD8 depletion can be effective in suppressing postoperative cancer metastasis by recovering the anti-tumor immunity.

## Discussion

Despite effective in removing malignant solid tumors, recurrence and metastatic rates after surgical resection are still high [31]. Stress induced by surgery can prompt immune escape and progression of the tumor by triggering postoperative inhibition of the cell-mediated immunity, including decreasing the number and activity of Th1 cells, CTLs, and NK cells [32]. Meanwhile, it has been well documented that surgery profoundly increases the number of immunosuppressive cells such as myeloid-derived suppressor cells (MDSCs) and Tregs [33]. Clinically, the elevation of CD4^+^CD25^+^FOXP3^+^ Tregs in the peripheral blood has been found to be associated with a higher risk of tumor recurrence and a poor prognosis in breast cancer patients [34]. A robust anti-tumor immune response can prevent or eliminate postoperative recurrence following surgical resection. However, approaches aimed at reversing immunosuppression are also critical, especially when Tregs act as crucial regulator of the tumor-killing function of effector cells. To explore potential therapeutic and preventive strategies for inhibiting postoperative metastasis by targeting Treg-mediated immunosuppression, it is necessary to elucidate the mechanisms directing the functional alterations of Tregs induced by surgery stress. In the present study, we, for the first time, identify NEDD8 as a major driver of Treg suppressive function in the context of surgical stress, and disclose the therapeutic potential of NEDD8 blockade to ameliorate surgical stress-facilitated lung metastasis in mice with colon cancer.

NEDD8 performs its biological function by conjugating to its target proteins, a process known as neddylation. NEDD8 conjugation / neddylation is catalyzed by three enzymes termed NEDD8-activating enzyme (NAE1), NEDD8-conjugating enzyme (E2), and NEDD8-ligating enzyme (E3) [35]. In recent years, the role of NEDD8-driven neddylation in tumor development has drawn considerable attention. The overactivated neddylation pathway was reported to contribute to the progression of multiple types of human cancers [36–38]. Furthermore, overexpression of NEDD8 is observed in cancer cells and causing aberrant proliferation, apoptosis, or migration [39, 40]. Targeting the neddylation pathway is hence, an emerging therapeutic strategy for cancers, inhibitors targeting different phases of this pathway have been investigated in previous and recent cancer research. For example, MLN4924 inhibiting NAE1 activity exhibits cytotoxic activity against a variety of human tumor-derived cell lines [41], induced apoptosis or senescence in human lymphoma cells [42]. WS-383 blocking the Dcn-Ubc12 interaction induces the accumulation of tumor suppressors p21, p27, and NRF2 [43]. However, whether neddylation also plays a part in postoperative cancer recurrence and metastasis is an intriguing question. Here, evidenced by the amelioration of lung metastasis in NEDD8 knock-out mice after laparotomy surgery, we propose a sound rationale for the involvement of NEDD8 in surgical stress-induced metastasis and NEDD8 blockade as an attractive anti-postoperative metastasis strategy.

As discussed above, the role of NEDD8-mediated neddylation in tumor progression has already been recognized. These studies, however, primarily focused on its direct effects of cancer cells. Growing but insufficient studies are exploring the influences of neddylation inhibition on the function of other important components of the tumor microenvironment, including immune cells (mainly macrophages [44, 45], cancer-associated fibroblasts [46] and cancer-associated endothelial cells [47]. Nevertheless, there is scarce evidence on the role and regulatory mechanism of NEDD8 in Tregs. It was, therefore the aim of our study to identify a missing link between NEDD8/neddylation and Tregs, specifically in the context of surgical stress-induced metastasis. Upon surgical stress, the expression of NEDD8 was found upregulated in Tregs, accompanied by the enhanced suppressive function of Tregs on CD8^+^T cells and NK cells. In contrary, NEDD8 depletion remarkably suppressed the function of Tregs. Our data also showed that NEDD8 deletion alone increased the frequency of CD8^+^T cells and NK cells, which indicates enhanced anti-tumor activity. Although we can’t exclude the direct impact of NEDD8 on these cells, we can conclude that NEDD8 depletion increase the number of these cells partially through Treg, as shown by the impaired Treg inhibitory function on CD8^+^T cell and NK cells.

Beyond cullins, the first protein family known as NEDD8 substrates, an increasing number of non-cullin proteins are found to be neddylated [5]. Among which, PINK1/Parkin, a critical pathway of mitophagy, was recognized as target of NEDD8 covalent conjugation [48], indicating the potential involvement of NEDD8-mediated neddylation in mitophagy regulation. Mitochondrial dynamics play an important role in immune responses mediated by various cell types [49]. As a selective autophagic process to clear aged and/or damaged mitochondria, mitophagy can be induced by multiple stresses [50] and is closely related to various cellular processes, including the development, activation, differentiation, or senescence of T cells [51, 52]. Previous studies have demonstrated that functional alterations of Tregs are associated with dysfunctional mitophagy [29, 53], but none have explored the underlying regulatory network, especially in tumor or the surgical stress microenvironment.

In our study, we observed mitophagy induced by surgical stress and inhibited by NEDD8 knock out. However, only the expression level of Parkin was increased and affected by NEDD8 knock out but not PINK1. Although PINK1 and Parkin are well-established synergistic mediators of mitophagy, Parkin seems to have the potential to activate mitophagy independently of PINK1. Whether it occurs during surgical stress-induced metophagy needs to be investigated. When NEDD8/neddylation was blocked, dysfunctional mitophagy resulted in the impaired immunosuppressive function of Tregs. Based on these findings, we conclude that NEDD8/neddylation is a crucial regulatory mechanism of elevated Treg immunosuppression upon surgical stress, which leads to defective anti-tumor immunity and, finally the postoperative metastasis. What awaits to be elucidated is how NEDD8 is upregulated in Tregs upon surgical stress.

In conclusion, by exploring the role and mechanisms underline Tregs functional alterations upon surgical stress, we demonstrate that surgical stress upregulated NEDD8 expression is essential for the immunosuppressive function of Tregs and contributes to the metastasis of colon cancer in mice. NEDD8/neddylation blockade may break Treg-mediated immune tolerance, restore the anti-tumor response and improve prognosis of cancer patients after surgery.

### Supplementary information

Supplementary Table

Supplementary Raw Data

Reproducibility checklist

## Data Availability

The data that support the findings of this study are available from the corresponding author upon reasonable request.
