# Supplementary material for: Targeting NEDD8 suppresses surgical stress-facilitated metastasis of colon cancer via restraining regulatory T cells
Source: Cell Death Dis. 2024 Jan 5;15(1):8. doi: 10.1038/s41419-023-06396-6 (PMC10767093; doi:10.1038/s41419-023-06396-6)
Supplement: Supplementary file 1 — Supplementary Table [file 41419_2023_6396_MOESM1_ESM.docx]

**Supplementary Table 1. List of primers used in quantitative real-time PCR.**

| **Name** | **Forward (5’-3’)** | **Reverse (5’-3’)** |
| --- | --- | --- |
| NEDD8 | GACGCTGACCGGAAAGGAGA | GAGCCTCTGCTGTTGTGGGG |
| Parkin | GTCGATGAAAGAGCCGCCGA | CAGCCACAGTTCCAGCACCA |
| Foxp3 | GAGGCTCCAGAGAAGCAGCG | TCCAGCTCATCCACGGTCCA |
| GAPDH | GCGGGGCTCTCCAGAACATC | TCCACCACTGACACGTTGGC |

**Supplementary Table 2. List of antibodies.**

| **Name** | **Cat. #** | **Manufacturer** |
| --- | --- | --- |
| Anti-Nedd8 antibody | ab4751 | Abcam |
| Anti-Parkin antibody | 4211 | Cell Signaling Technology |
| Anti-Pink1 antibody | 6946 | Cell Signaling Technology |
| Anti-Nix antibody | 12396 | Cell Signaling Technology |
| Anti-BNIP3 antibody | 44060 | Cell Signaling Technology |
| Anti-Drp1 antibody | 8570 | Cell Signaling Technology |
| Anti-FUNDC1 antibody | 49240 | Cell Signaling Technology |
| HRP-linked Anti-mouse IgG | 7076 | Cell Signaling Technology |
| HRP-linked Anti-Rabbit IgG | 7074 | Cell Signaling Technology |
| HRP-linked Anti-GAPDH antibody | 8840 | Cell Signaling Technology |
