# Supplementary figures and images for: Targeting NEDD8 suppresses surgical stress-facilitated metastasis of colon cancer via restraining regulatory T cells
Source: Cell Death Dis. 2024 Jan 5;15(1):8. doi: 10.1038/s41419-023-06396-6 (PMC10767093; doi:10.1038/s41419-023-06396-6)

Figure 1G

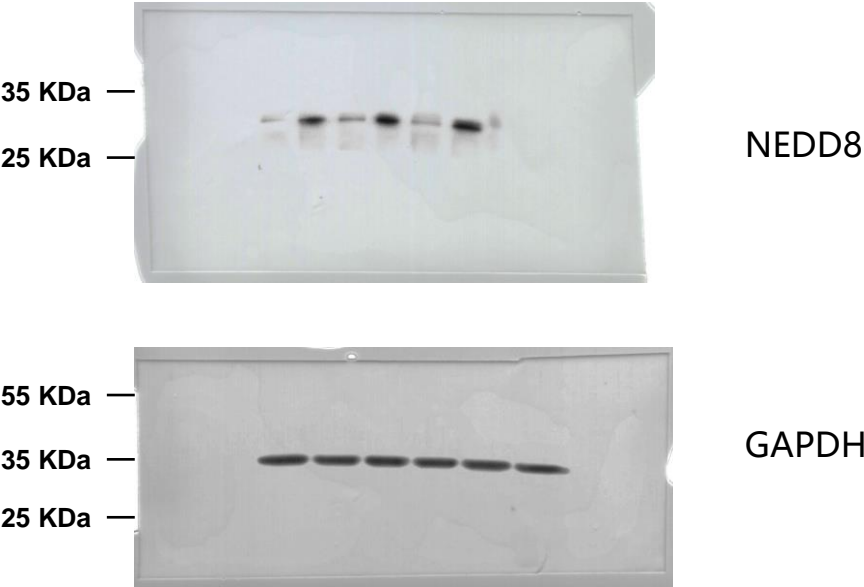

Figure 6F

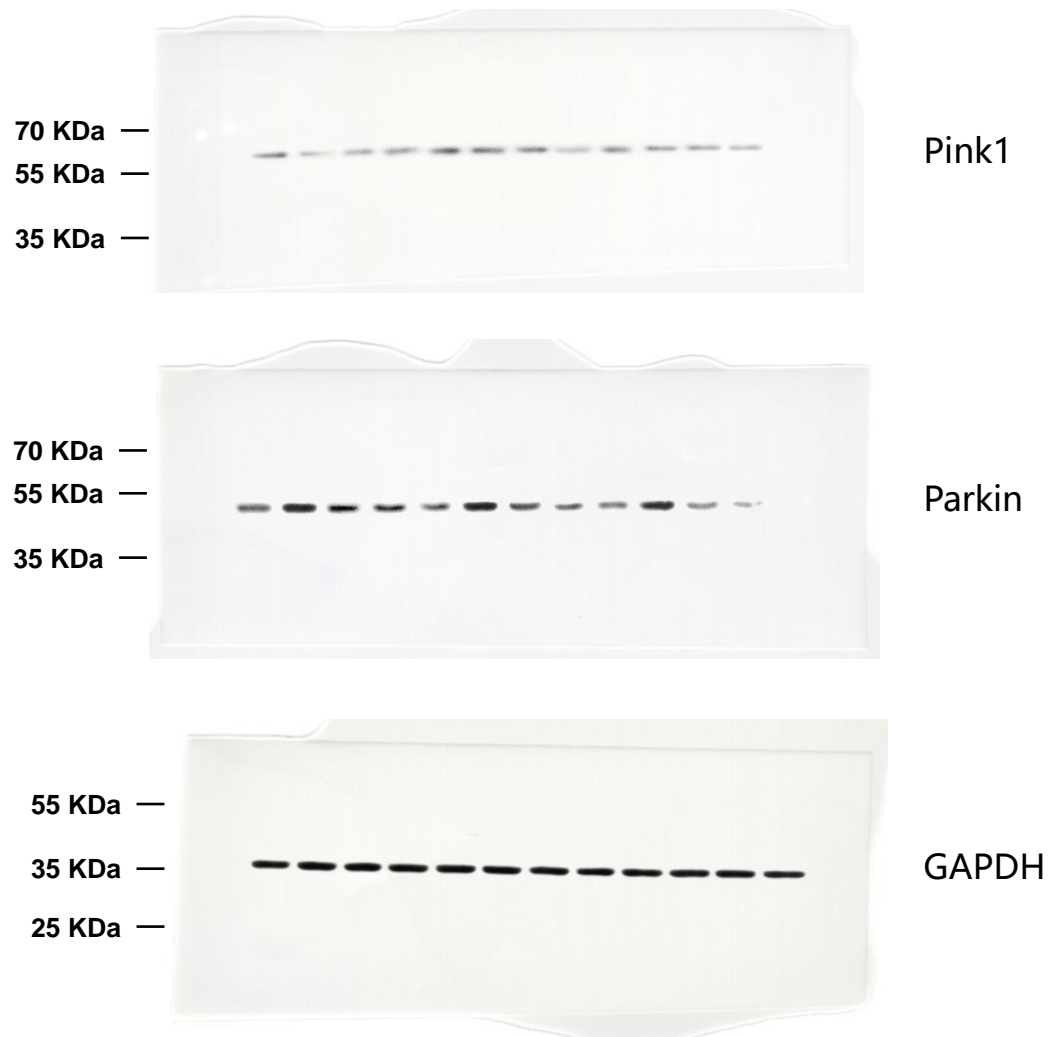

Figure 6G

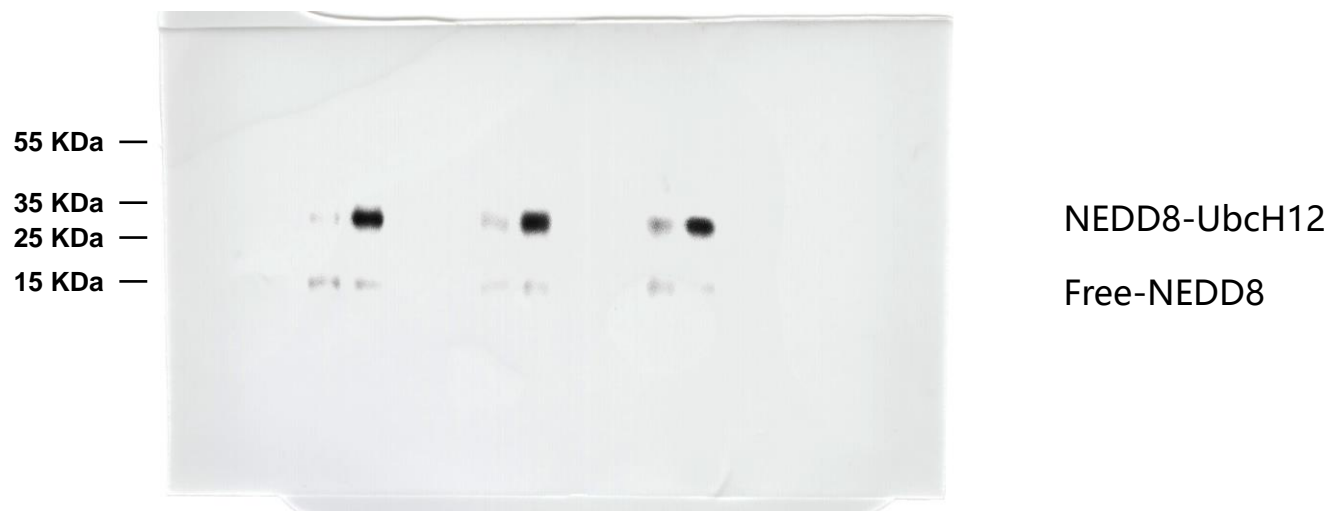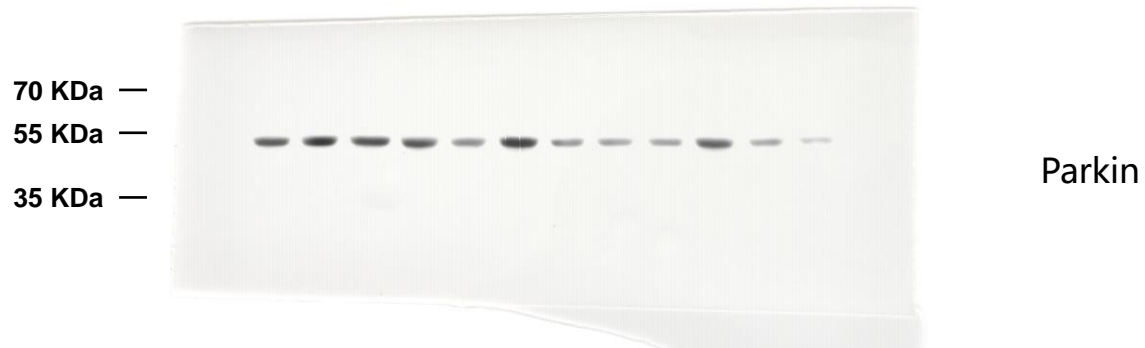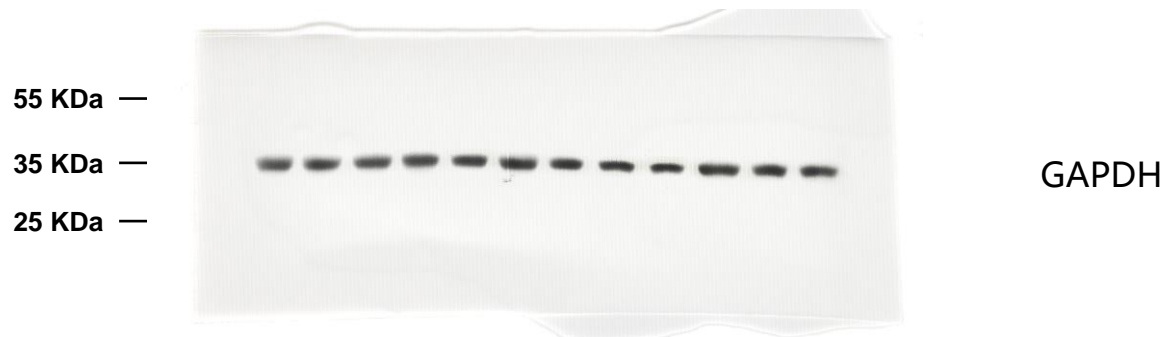

Figure 6H

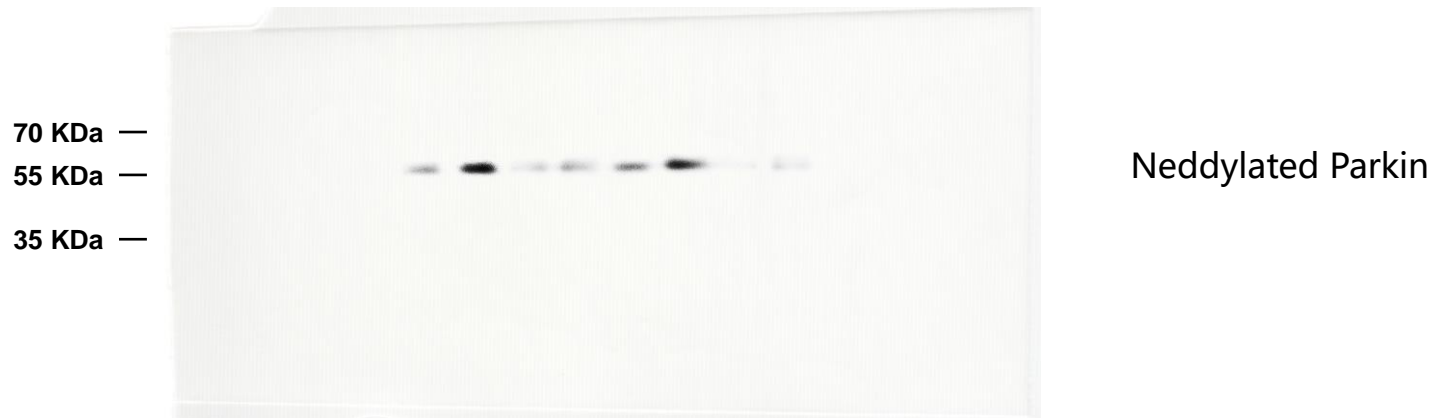

Figure 6F

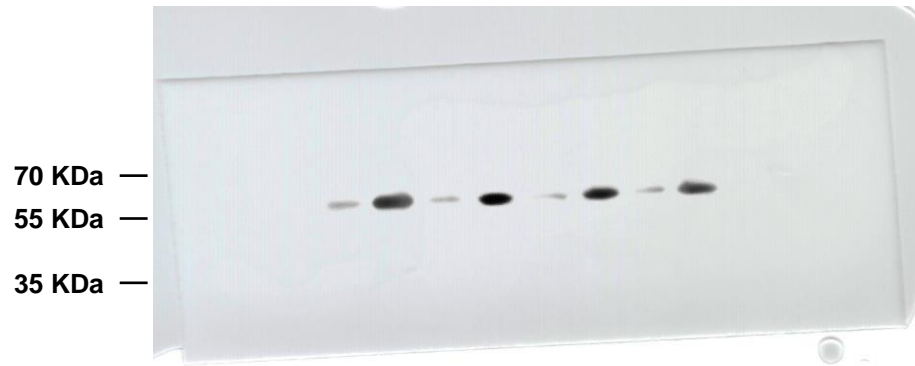

IB: Pink1

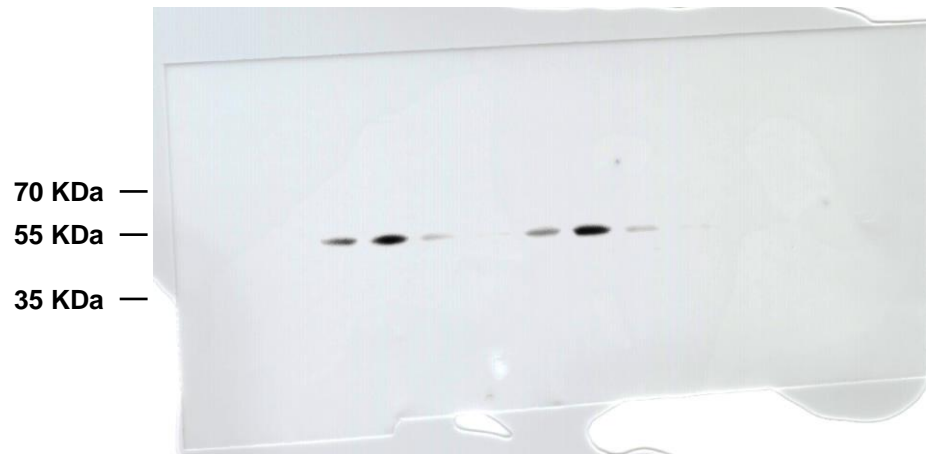

IB: Parkin

Supplement: Supplementary file 2 — Supplementary Raw Data [file 41419_2023_6396_MOESM2_ESM.pdf]
